# Toxic Effects of Size-tunable Gold Nanoparticles on *Caenorhabditis elegans* Development and Gene Regulation

**DOI:** 10.1038/s41598-018-33585-7

**Published:** 2018-10-15

**Authors:** Chun-Chih Hu, Gong-Her Wu, Sheng-Feng Lai, Muniesh Muthaiyan Shanmugam, Y. Hwu, Oliver I. Wagner, Ta-Jen Yen

**Affiliations:** 1 Department of Materials Science and Engineering, National Tsing Hua University, Hsinchu, 30013 Taiwan; 2 Department of Life Science and Institute of Molecular & Cellular Biology, National Tsing Hua University, Hsinchu, 30013 Taiwan; 3 Institute of Physics, Academia Sinica, Taipei, 115 Taiwan

## Abstract

We utilized size-tunable gold nanoparticles (Au NPs) to investigate the toxicogenomic responses of the model organism *Caenorhabditis elegans*. We demonstrated that the nematode *C*. *elegans* can uptake Au NPs coated with or without 11-mercaptoundecanoic acid (MUA), and Au NPs are detectable in worm intestines using X-ray microscopy and confocal optical microscopy. After Au NP exposure, *C*. *elegans* neurons grew shorter axons, which may have been related to the impeded worm locomotion behavior detected. Furthermore, we determined that MUA to Au ratios of 0.5, 1 and 3 reduced the worm population by more than 50% within 72 hours. In addition, these MUA to Au ratios reduced the worm body size, thrashing frequency (worm mobility) and brood size. MTT assays were employed to analyze the viability of cultured *C*. *elegans* primary neurons exposed to MUA-Au NPs. Increasing the MUA to Au ratios increasingly reduced neuronal survival. To understand how developmental changes (after MUA-Au NP treatment) are related to changes in gene expression, we employed DNA microarray assays and identified changes in gene expression (e.g., clec-174 (involved in cellular defense), cut-3 and fil-1 (both involved in body morphogenesis), dpy-14 (expressed in embryonic neurons), and mtl-1 (functions in metal detoxification and homeostasis)).

## Introduction

During the rapid development of nanotechnology, nanomaterials have attracted considerable attention because of their size-dependent optical and electrical properties. A negative side effect, however, is their release into soil and water, likely posing a considerable risk to human health^1,2^. Gold nanoparticles (Au NPs) belong to a biocompatible class of nanomaterials widely used for bioimaging^3,4^, biosensing^5,6^, facial creams^7^, and targeted therapeutic purposes^8–10^. Nevertheless, whether these nanoparticles can be directly taken up by animals, affecting their physiology and thus posing an ecotoxicological risk, remains unclear. Only a few recent studies have investigated the possible environmental effects of Au NPs^2,11^. Based on the quantum effect properties, Au NPs less than 2 nm in size are also utilized as fluorescent probes for bioimaging, meaning that these NPs can be taken up by cells, potentially affecting their viability^12^. In our study, we utilized a protocol to rapidly synthesize Au NPs with an 11-mercaptoundecanoic acid (MUA) coating, allowing us to control the particle size (via X-ray irradiation)^13^. *C*. *elegans* has been widely used to elucidate the toxicological effects of metal NPs on higher organisms^14–18^. However, whether Au NPs less than 1 nm in particle size (e.g., used in bioimaging) directly affect neuronal development, and in turn, the locomotion behavior of animals remains unclear. The importance of *C*. *elegans* in toxicology studies stems from its small size (convenient to maintain on agar plates), short life cycle (approximately 2.5 days)^19^, and fully sequenced genome^20^, which are all advantageous for genetic studies. Furthermore, changes in worm locomotion behavioral assays can be related to altered motor neuronal function, which is important for understanding neurological diseases, such as amyotrophic lateral sclerosis (ALS) and Parkinson’s disease^21–23^. Thus, we chose to employ nematodes to evaluate the toxicity of Au NPs. Notably, the uptake and toxicity of Au NPs highly depends on their shape^24^, size^25^ and surface charge^26,27^ and these studies revealed that Au NPs can accumulate in cells. However, how very small (<1 nm) Au NPs (e.g., used for bioimaging) affect neuronal viability, in turn affecting motor neuron function (indicated by changes in animal mobility), remains unclear. Critically, primary neurons can be easily isolated from *C*. *elegans* embryos and cultured for several days at room temperature without adjusting the CO_2_ atmosphere. This straightforward protocol enables the determination of developmental changes in neurons (e.g., axon growth) upon MUA-Au NP exposure. We also investigated how Au NP uptake affects gene regulation in nematodes by employing commercially available DNA microarray gene chips. With this assay, changes in the expression of 22,548 *C*. *elegans* genes (with high homology to human genes) were detected by comparing control animals and Au NP-treated animals.

## Results and Discussion

### Characterization of Au NPs

**Figure 1:**
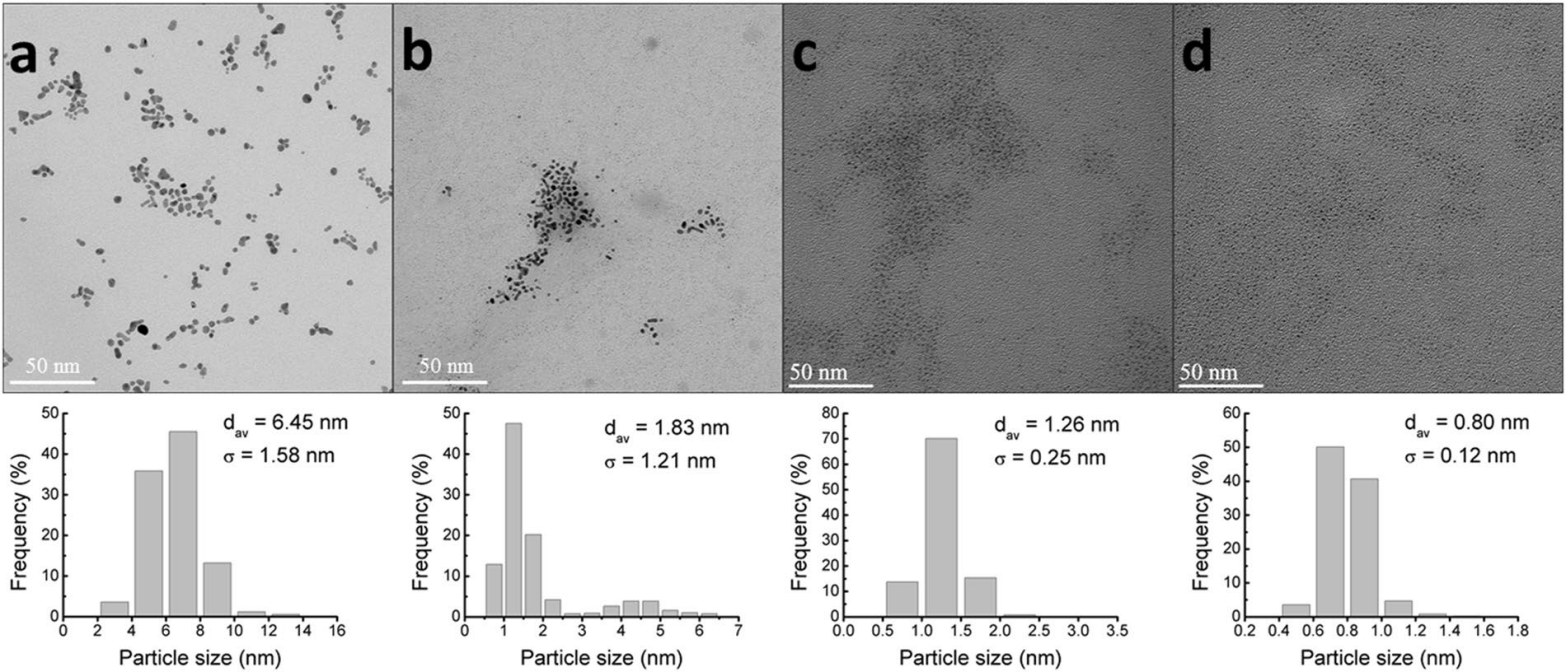
TEM micrographs of Au NPs synthesized in the absence of MUA and in the presence of increasing MUA concentrations and the corresponding size histograms (n > 500). Large, nonspherical NP results are shown with no MUA present (**a**). With increasing MUA to Au ratios of 1:2 (**b**), 1:1 (**c**), and 3:1 (**d**), the NP dimensions and standard deviations decreased. Inset: size distribution of Au NPs.

**Figure 2:**
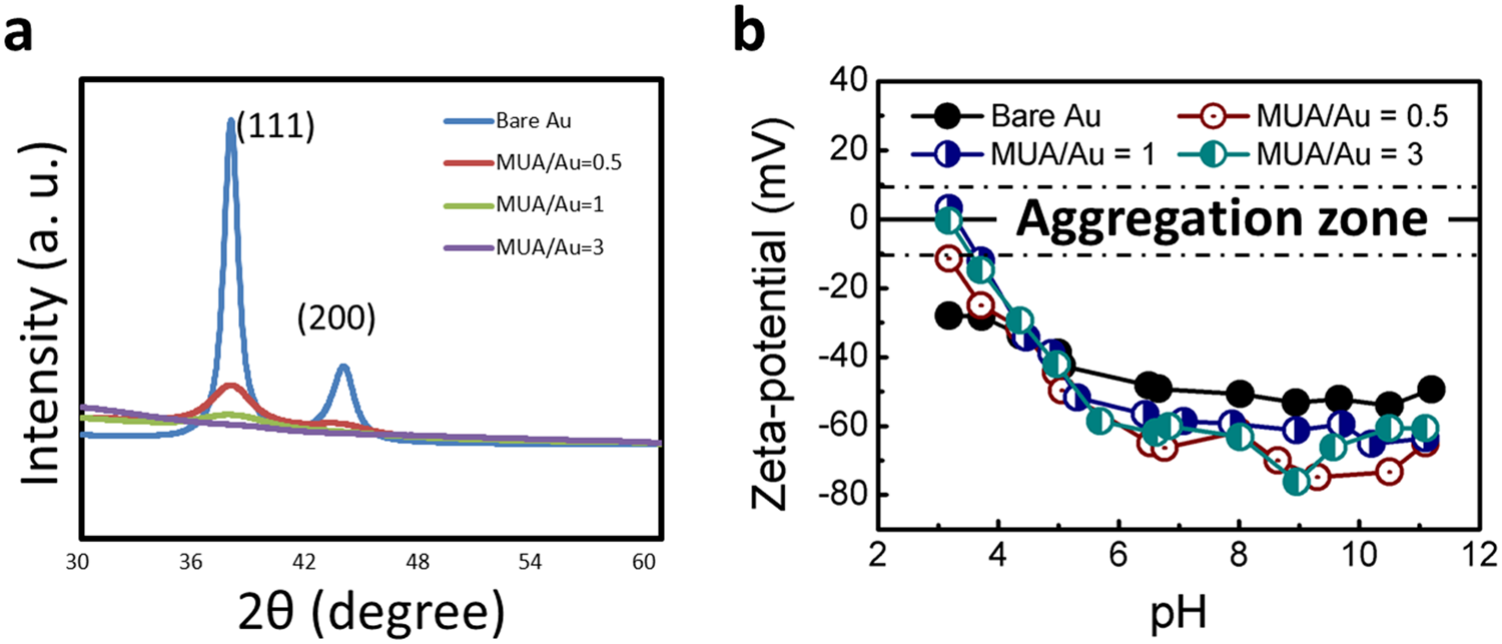
(**a**) XRD patterns of Au NP colloidal solutions obtained with and without MUA. The two peaks corresponded to 111 and 200 metallic gold planes and were visibly narrower when MUA was absent from the precursor solution, indicating that larger particles were formed without MUA than with MUA. (**b**) Zeta potential measurements of Au NP colloidal solutions obtained with and without MUA. High zeta potential (either positive or negative, >10–15 mV) led to monodispersity.

Bare Au and MUA-Au NPs (exhibiting different particle sizes) were synthesized by intense X-ray irradiation as described previously^13^. This method provides a simple and rapid procedure to reduce Au ions into Au NPs and further control the size of MUA-Au NPs produced by X-ray irradiation^13^. Thus, by controlling the MUA to Au ratio, we tuned the size of Au NPs from 6.45 nm to 0.8 nm, as shown in Fig. 1. Note that MUA plays an important role in terminating the nucleation and growth of Au NPs due to its high affinity for Au. The process of reducing Au ions into Au NPs occurs in the subsecond range and is independent of the MUA concentration, allowing accurate particle size control. The particle sizes of bare Au and MUA-Au NPs with MUA to Au ratios of 0.5, 1, and 3 were 6.45 ± 1.58, 1.83 ± 1.21, 1.26 ± 0.25, and 0.80 ± 0.12 nm, respectively (Table 1). The presence of MUA (leading to small-sized particles) was verified by X-ray diffraction (XRD) analysis. The XRD peaks in Fig. 2a corresponding to 111 and 200 planes of metallic gold were clearly narrower in the absence of MUA, suggesting the formation of larger particles compared to those formed in the presence of MUA. The zeta potential measurements in Fig. 2b and Table 1 indicated that bare Au and MUA-Au NPs were well dispersed in S medium because of high zeta potential (either positive or negative, >10–15 mV), leading to monodispersity.

**Table 1:** Characteristics of the Au NPs used as determined by TEM and zeta potential.

Sample	Diameter (nm ± STD)	Number of particles analyzed	Zeta potential at pH 6.5 (mV)
Bare Au	6.45 ± 1.58	>500	−49.2
MUA/Au = 0.5	1.83 ± 1.21	>500	−65
MUA/Au = 1	1.26 ± 0.25	>500	−56.6
MUA/Au = 3	0.80 ± 0.12	>500	−61.9

### Bare Au and MUA-Au NP Uptake by *C*. *elegans*

**Figure 3:**
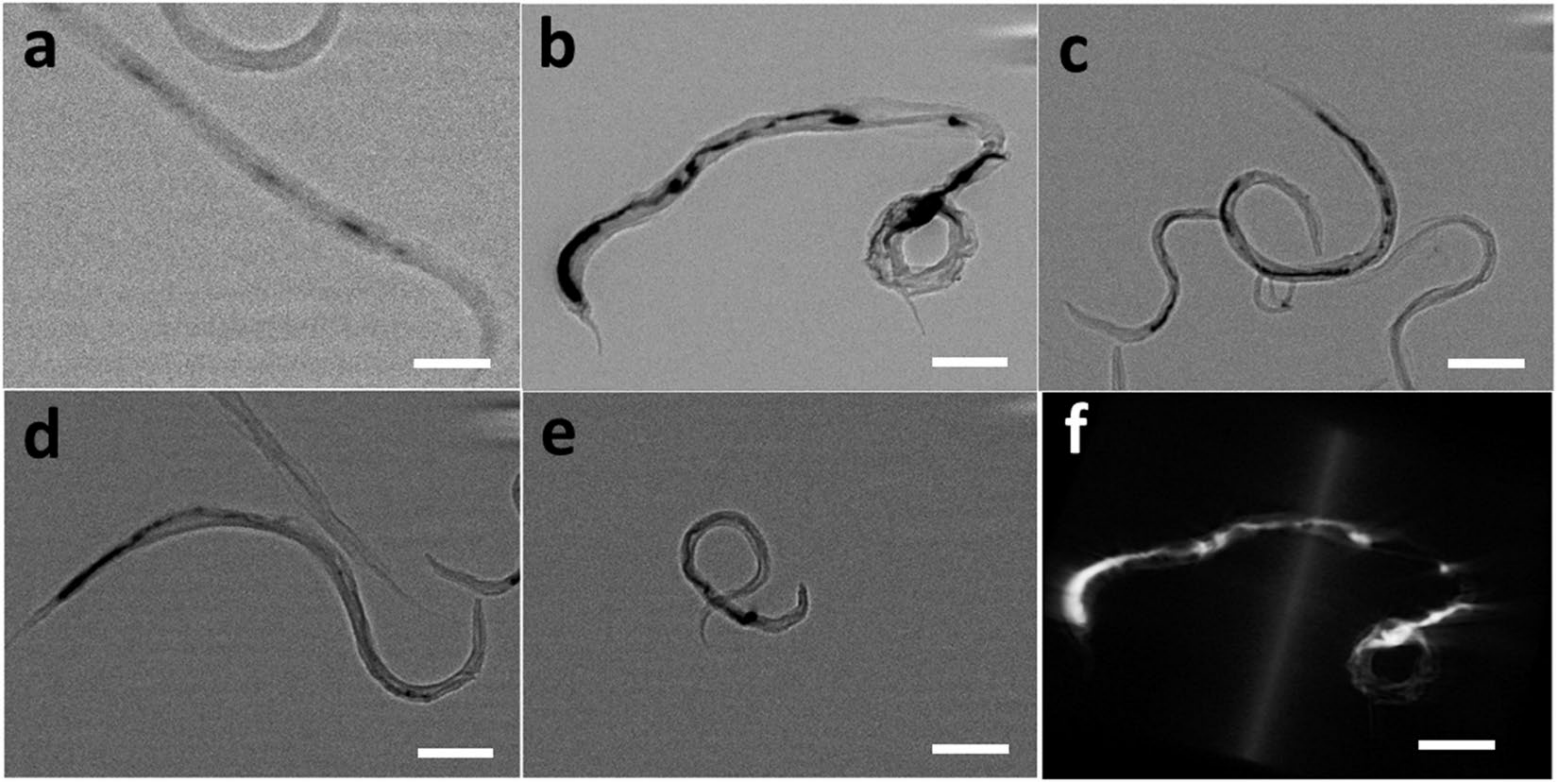
High-resolution X-ray micrographs of bare Au and MUA-Au NPs in the *C*. *elegans* intestine. (**a**) 2D high-resolution X-ray micrographs in the absence of Au NPs. (**b**) 2D high-resolution X-ray micrographs of bare Au NPs. (**c**) 2D high-resolution X-ray micrographs with an increasing MUA: Au ratio of 1:2. (**d**) 2D high-resolution X-ray micrographs with an increasing MUA: Au ratio of 1:1. (**e**) 2D high-resolution X-ray micrographs with an increasing MUA: Au ratio of 3:1. (**f**) 3D reconstruction of the micrographs in (**b**). After incubating worms with bare Au NPs for 12 hours, aggregation was found in the worm intestines, demonstrating that bare Au NPs can be taken up by *C*. *elegans*. Scale bars: 100 µm.

Worms were incubated with bare Au or MUA-Au NPs overnight and subsequently imaged using X-ray microscopy to determine if and how Au NPs are ingested by worms. Au NPs are likely incapable of penetrating the highly impermeable worm cuticles^28,29^ and we assume that Au NPs are taken up by the mouth (pharynx) of the animal. Critically, the internalization of various NPs rather than the NP exposure concentration was previously demonstrated to be a toxicological indicator^18,30^. Therefore, visualizing Au NPs in worm intestines is very important, as shown in Fig. 3. Notably, while we employed X-ray microscopy to visualize bare Au and MUA-Au NPs in the worm intestines, other groups used X-ray fluorescence^31^ as well as dark-field hyperspectral imaging^32^ to visualize metal NPs in worms. The uptake of bare Au and MUA-Au NPs into worm tissues were confirmed by confocal microscopy imaging using FITC-labeled NPs (Supplementary information).

### Determination of Toxicity Endpoints

**Figure 4:**
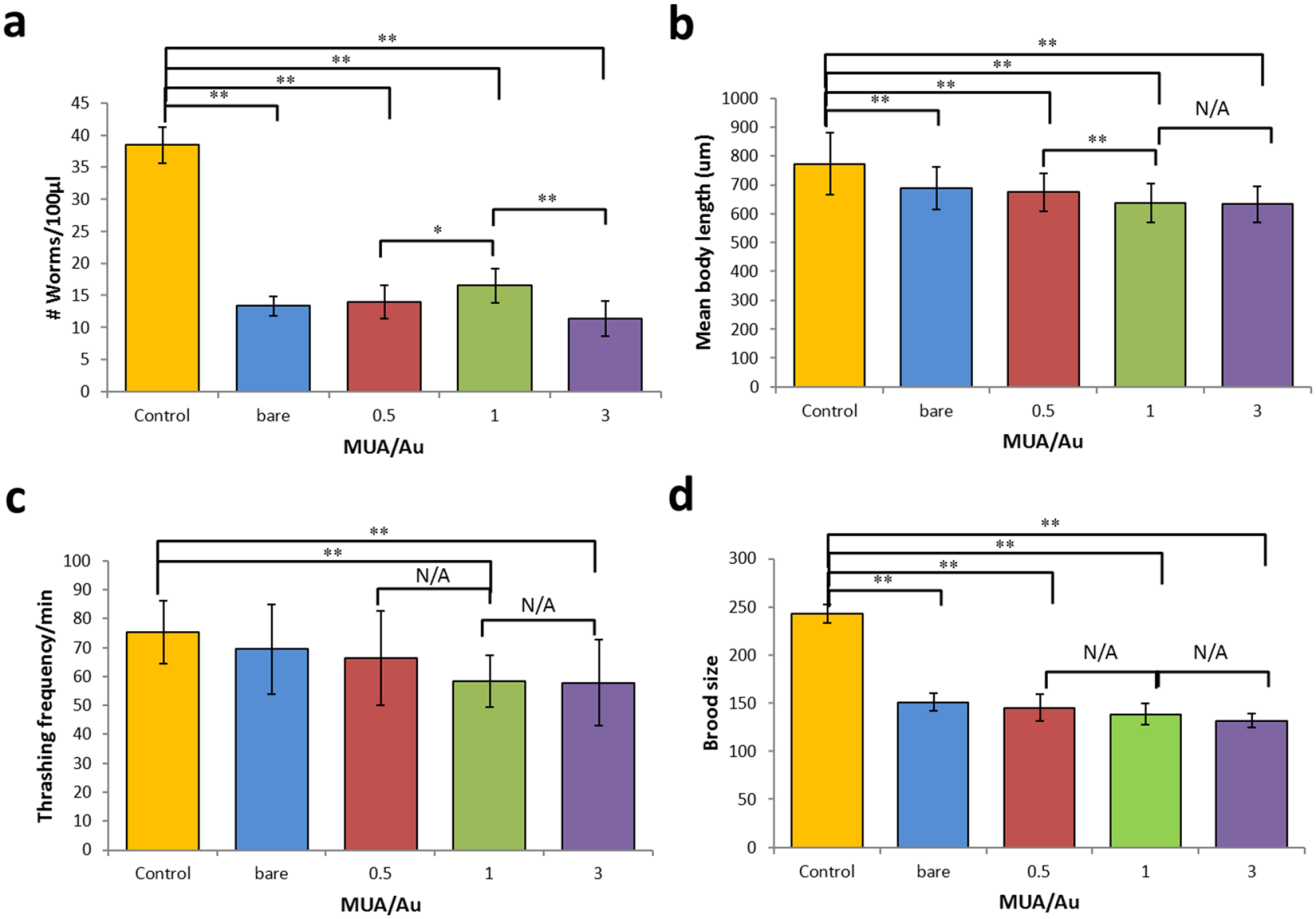
Effect of Au NPs with and without MUA on (**a**) worm population, (**b**) worm body length, (**c**) worm locomotion (worm thrashing assay) and (**d**) brood size. T-test: *p < 0.05, **p < 0.01. Error bars: ± SD.

Critical endpoints in toxicology, such as body length, locomotion, reproduction (changes in population), and brood size, have been widely investigated in previous studies^18,33^. Various conditions, such as exposure time^15^, surface modification^34^, particle size^11^, and Au NP concentration^17^, are used to determine the toxicity of Au NPs. Moon *et al*.^15^ utilized *C*. *elegans* to investigate the multigenerational effects of Au NPs after continuous and intermittent food intake and found that continuous exposure yielded significantly different worm phenotypes. Yang *et al*.^11^ mentioned that different Au particle sizes accumulate in different mouse organs and that 4.5-nm Au NPs are excreted through the urine of pregnant mice, while 30-nm Au NPs exhibit longer blood circulation times. Consequently, Au NPs of smaller sizes were easily excreted from the body. Notably, for the first time, this study evaluated ultra-small Au NPs (0.80 ± 0.12 nm), similar to those used for bioimaging, and revealed the physiological, genetic, and behavioral consequences on a nematode. Crucially, Matulionyte *et al*.^34^ indicated that different surface modifications of Au NPs resulted in different cytotoxicities. Thus, we employed MUA coating to further fine-tune the Au NP size and to determine toxicity endpoints, such as body length, locomotion, reproduction and brood size, which are all primary indicators of toxicity. Figure 4a shows that all four of the tested conditions (worms treated with bare Au or MUA-Au NPs with MUA to Au ratios of 0.5, 1 or 3) significantly reduced the worm population by more than 50%, while the strongest effect was found for the MUA to Au ratio of 3, reducing the population by 70.39%. Similarly, MUA-Au NP size-dependent effects were observed in mean body length reductions (Fig. 4b), suggesting that bare Au and MUA-Au NPs exert adverse effects on worm growth. Interestingly, the locomotion of the worms was only affected at particle sizes smaller than 1.83 ± 1.21 nm (Fig. 4c and Table 1). For the brood size assay, all MUA-Au NP ratios (including bare Au) significantly decreased the number of eggs laid (Fig. 4d), suggesting that these NPs largely reduced worm fertility. The average brood size for each untreated control worm was 250, while this value was reduced to 150 eggs on average after Au NP exposure (60%).

### Effects of MUA-Au NPs on Neuronal Cell Viability

**Figure 5:**
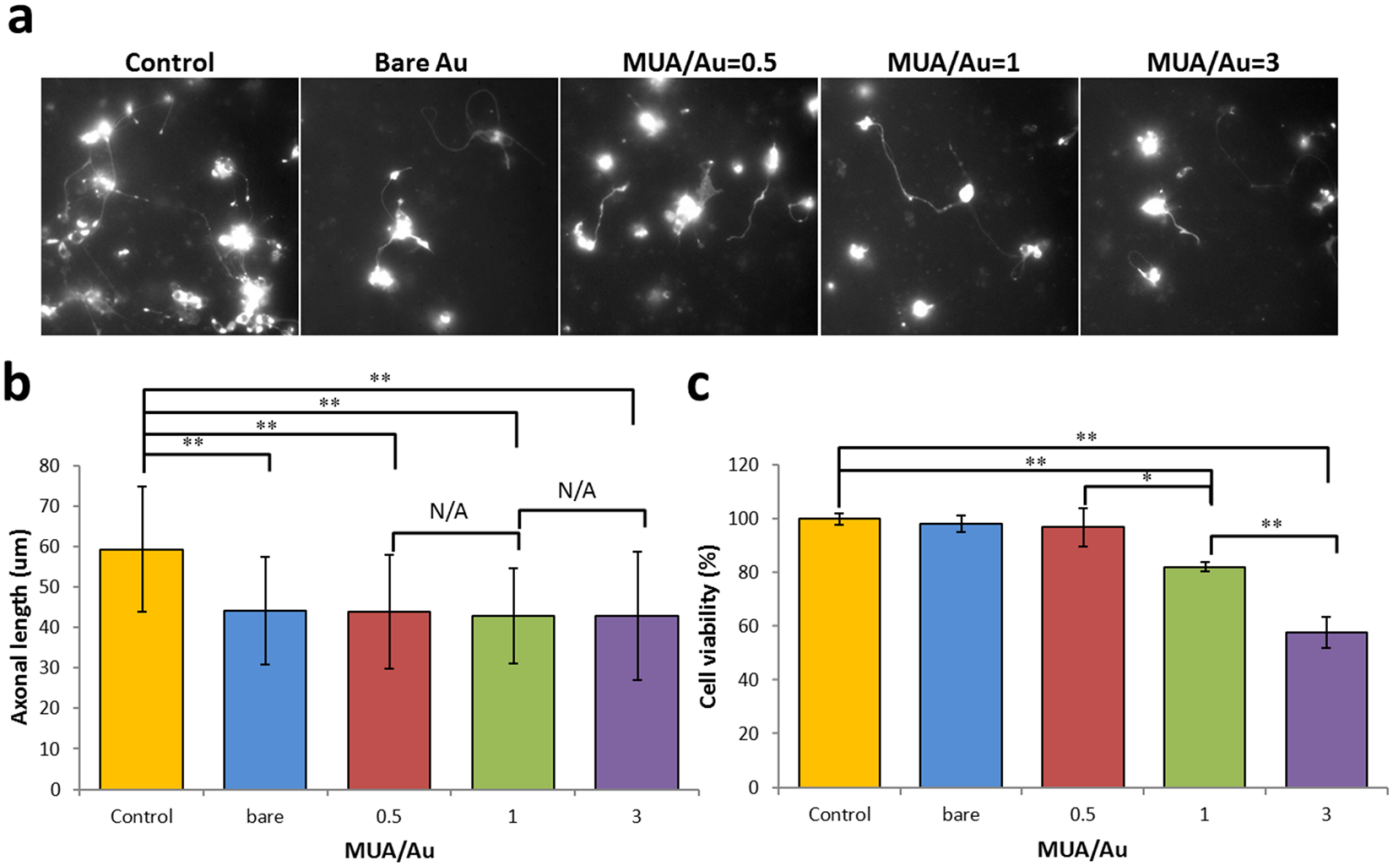
(**a**,**b**) Images from primary neuronal cells in culture (expressing an mRFP-tagged axonal marker) reveal adverse effects on axonal growth upon exposure to Au NPs. (**c**) Cellular viability of neurons exposed to Au NPs indicated that small Au NPs reduced neuronal cell viability. T-test: *p < 0.05, **p < 0.01. Error bars: ± SD.

We next wanted to assess whether the effects of MUA-Au NP uptake are beneficial or detrimental to the development of primary cultured *C*. *elegans* neurons. One study demonstrated that Au NPs have beneficial effects on the differentiation, growth, and maturation of neurons^35^, while another study reported adverse cellular effects of Au NPs^2^. Since we revealed significant changes in worm locomotion upon exposure to MUA-Au NPs (Fig. 4c), we hypothesized that this effect is based on impeded motor neuron function^21–23^. Therefore, we examined primary neurons isolated from *C*. *elegans* embryos before and after Au NP exposure and found that axonal growth was significantly impeded by exposure to both bare Au and MUA-Au NPs (Fig. 5a,b). Interestingly, while this effect was independent of the MUA to Au ratio, the effect on cell viability (using the MTT assay) was dependent on this ratio, indicating that increasing the MUA to Au ratio also decreases the neuronal survival rate (Fig. 5b,c). Importantly, cell viability was decreased at only Au NP particle sizes equal to or smaller than 1.26 ± 0.25 (Fig. 5c and Table 1), which was consistent with a study from another group revealing similar results on the onset of cell death versus Au particle size^36^. Smaller particles are likely capable of triggering higher oxidative stress on cells than larger particles because of their extensive surface areas^33^.

### *C*. *elegans* Gene Expression After Au NP Exposure

**Figure 6:**
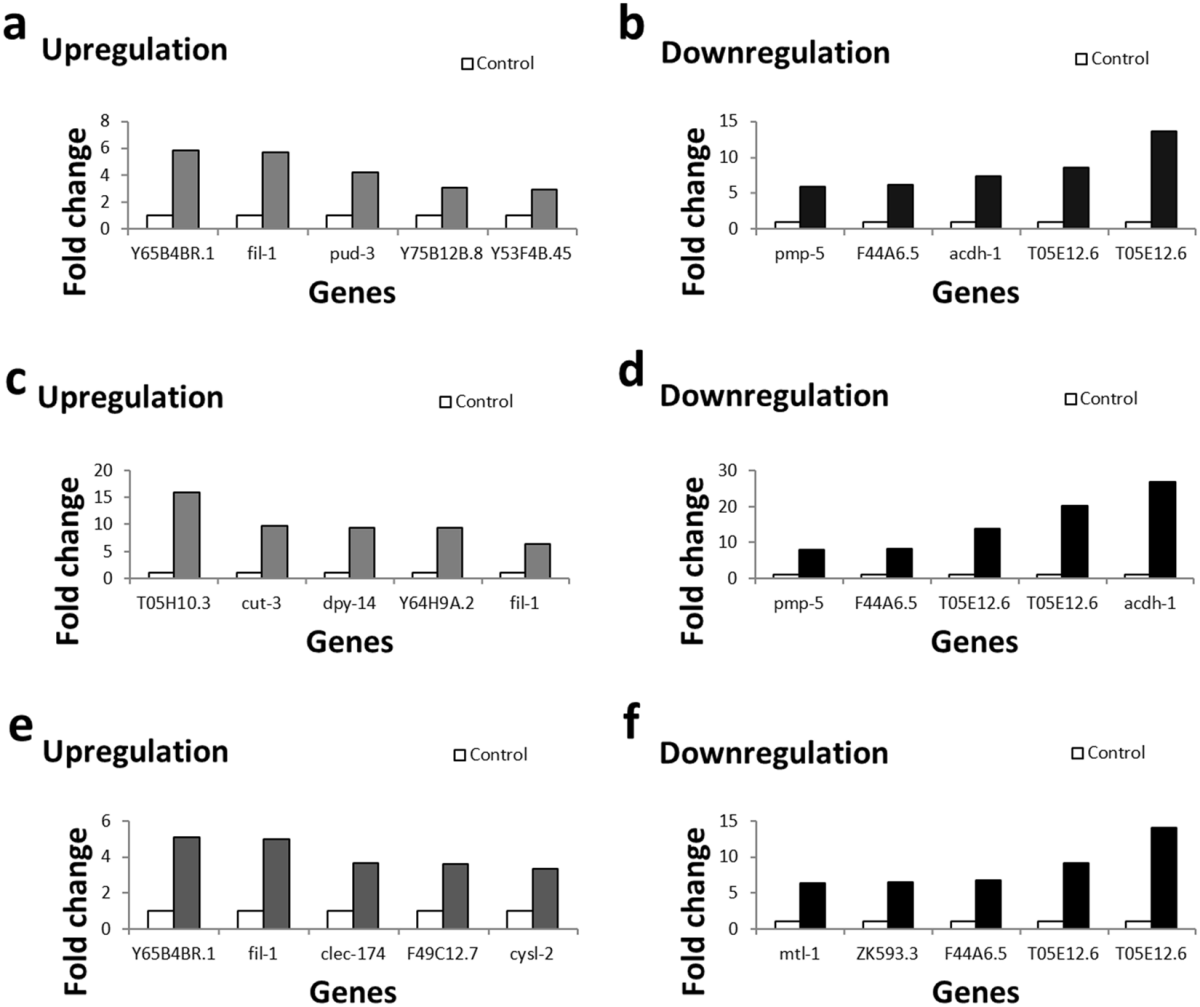
DNA microarray results from worms exposed to bare Au NPs. (**a**) Examples of genes upregulated 3–6-fold. (**b**) Examples of genes downregulated 6–13-fold. DNA microarray results from worms exposed to MUA-Au NPs with MUA/Au NPs = 0.5. (**c**) Examples of genes upregulated 6-16-fold. (**d**) Examples of genes downregulated 8-27-fold. DNA microarray results from worms exposed to MUA-Au NPs with MUA/Au NPs = 3. (**e**) Examples of genes upregulated 3-5-fold. (**f**) Examples of genes downregulated 6-14-fold. For details on the cellular functions of these genes, refer to Tables 2–4.

To understand how our observations on worm locomotion, brood size and neuronal development (Figs 4 and 5) relate to cellular mechanisms, specifically at the genetic level, we employed DNA microarray assays. As a result, 197 genes were differentially expressed after exposing worms to bare Au, 191 genes were differentially expressed after exposure to MUA/Au = 0.5, and 112 genes were differentially expressed after exposure to MUA/Au = 3. The 10 critical genes shown in Fig. 6 (Tables 2–4) are involved in defense response, lipid catabolic processes, lipid storage, lifespan, body morphogenesis, regulation of body shape (and size), metal detoxification (and homeostasis) and stress adaptation. Some of these genes revealed similar functions in behavioral and cellular regulations. Y65B4BR.1 and clec-174, both of which are involved in cellular defense responses critical for eliminating intruders, such as bacteria, viruses and other particles (likely including NPs), are worth mentioning. ZK593.3 and fil-1 are both involved in lipid storage and are thus related to the regulation of body shape and size (Tables 3 and 4). Additionally, the identified gene cut-3 is involved in body morphogenesis, and dpy-14 also regulates body shape and size; therefore, all these genes may be related to the observed decreased mean body length (Fig. 4b). Interestingly, loss of acdh-1 activity (one of the identified genes) can shorten the lifespan and may thus be related to the observed decrease in population size upon Au NP exposure (Fig. 4a). mtl-1 is involved in both metal detoxification and worm growth and fertility, affecting the growth of a worm population. Importantly, various identified genes, such as Y53F4B.45, dpy-14 and F49C12.7, are expressed in neurons. Notably, another study (using a gene candidate screen, different from our forward genetic approach) identified various molecular chaperones (hsp-16.1, hsp-70, hsp-3, and hsp-4) involved in endoplasmic reticulum stress upon worm exposure to Au NPs^17^. Future work should focus on investigating how the identified genes relate to the observed behavioral defects at the molecular level. For example, mutant worms carrying knockout alleles in the various identified genes should be identified, and the molecular pathways and signaling events leading to the observed phenotypes (worm locomotion, population and body length) should be dissected. Further, in future studies, we must show whether overexpressing the identified genes rescues the observed phenotypic effects.

**Table 2:** Descriptions of the cellular functions of genes after exposure to bare Au NPs.

Gene expression after bare Au NP exposure	
***Upregulated genes***	
Y65B4BR.1	Y65B4BR.1 is an ortholog of human PLB1 (phospholipase B1); Y65B4BR.1 is involved in cellular defense response; Y65B4BR.1 is predicted to have hydrolase activity, acting on ester bonds, based on protein domain information.
fil-1	fil-1 is involved in lipid catabolic processes and lipid storage; fil-1 exhibits lipase activity; fil-1 is expressed in the intestine.
pud-3	pud-3 is expressed in the pm3, hyp7 syncytium, head, intestine, and rectal gland cells.
Y75B12B.8	Microarray studies indicate that Y75B12B.8 is regulated by clk-1, sir-2.1, and mir-243; RNA sequencing and microarray studies indicate that Y75B12B.8 is regulated by mianserin, quercetin, and single-walled carbon nanotubes.
Y53F4B.45	Microarray studies indicate that Y53F4B.45 is regulated by Cry5B, hydrolyzable tannins, humic substances, R24, selenium, sirolimus, and allantoin; RNA sequencing and microarray studies indicate that Y53F4B.45 is enriched in germ line cells, muscle cells and neurons, including DA neurons.
***Downregulated genes***	
pmp-5	pmp-5 is an ortholog of human ABCD4 (ATP-binding cassette subfamily D member 4); pmp-5 is predicted to have ATP-binding activity and ATPase activity, coupled to the transmembrane movement of substances, based on protein domain information; pmp-5 is expressed in the hypodermis and intestine.
F44A6.5	F44A6.5 encodes a novel protein conserved among nematodes.
acdh-1	acdh-1 encodes a short-chain acyl-CoA dehydrogenase; ACDH-1 is predicted to be a mitochondrial enzyme that catalyzes the first step of fatty acid beta-oxidation and thus plays a key role in energy production; steady-state acdh-1 mRNA levels are controlled by the MDT-15 mediator complex subunit; in addition, gene expression studies indicate that acdh-1 expression is upregulated in daf-2 mutant animals but downregulated in fasted animals; loss of acdh-1 activity via RNAi can shorten the lifespan.
T05E12.6	T05E12.6 is an ortholog of human EPHX1 (epoxide hydrolase 1); microarray and RNA sequencing studies indicate that T05E12.6 is regulated by heme, diallyl trisulfide, tunicamycin, D-glucopyranose, D-glucose, deoxyglucose, atrazine, fluoranthene, Cry5B, chlorpyrifos, diazinon, quercetin, humic substances, colistin, adsorbable organic bromine compounds, sirolimus, and Ag nanoparticles; RNA sequencing and microarray studies indicate that T05E12.6 is enriched in germ line cells, intestine, and I5, DA, SAB, and retrovesicular ganglia.
T05E12.6	T05E12.6 is an ortholog of human EPHX1 (epoxide hydrolase 1); microarray and RNA sequencing studies indicate that T05E12.6 is regulated by heme, diallyl trisulfide, tunicamycin, D-glucopyranose, D-glucose, deoxyglucose, atrazine, fluoranthene, Cry5B, chlorpyrifos, diazinon, quercetin, humic substances, colistin, adsorbable organic bromine compounds, sirolimus, and Ag nanoparticles; RNA sequencing and microarray studies indicate that T05E12.6 is enriched in germ line cells, intestine, and I5, DA, SAB, and retrovesicular ganglia.

Information taken from Wormbase (Wormbase.org). For additional details and citations, please refer to Wormbase.

**Table 3:** Descriptions of the cellular functions of genes after exposure to MUA/Au NPs = 0.5.

Gene expression after exposure to MUA/Au NP = 0.5	
***Upregulated genes***	
T05H10.3	T05H10.3 is expressed in the hypodermis.
cut-3	cut-3 is involved in body morphogenesis.
dpy-14	dpy-14 encodes a type III (alpha 1) collagen that is required for embryonic, larval, and vulval development, proper amphid morphology, and regulation of body shape and size; a dpy-14 promoter-GFP fusion construct is reportedly expressed in embryonic neurons.
Y64H9A.2	RNA sequencing and microarray studies indicate that Y64H9A.2 is regulated by methylmercury hydroxide, 1-methylnicotinamide, manganese chloride, D-glucose, fluoranthene, diazinon, chlorpyrifos, quercetin, paraquat, colistin, dafa #1, and sirolimus; microarray and tiling array studies indicate that Y64H9A.2 is enriched in germline precursor cells, the hypodermis and PVD and OLL neurons.
fil-1	fil-1 is involved in lipid catabolic processes and lipid storage; fil-1 exhibits lipase activity; fil-1 is expressed in the intestine.
***Downregulated genes***	
pmp-5	pmp-5 is an ortholog of human ABCD4 (ATP-binding cassette subfamily D member 4); pmp-5 is predicted to have ATP-binding activity and ATPase activity, coupled to the transmembrane movement of substances, based on protein domain information; pmp-5 is expressed in the hypodermis and intestine.
F44A6.5	F44A6.5 encodes a novel protein conserved among nematodes.
T05E12.6	T05E12.6 is an ortholog of human EPHX1 (epoxide hydrolase 1); microarray and RNA sequencing studies indicate that T05E12.6 is regulated by heme, diallyl trisulfide, tunicamycin, D-glucopyranose, D-glucose, deoxyglucose, atrazine, fluoranthene, Cry5B, chlorpyrifos, diazinon, quercetin, humic substances, colistin, adsorbable organic bromine compounds, sirolimus, and Ag nanoparticles; RNA sequencing and microarray studies indicate that T05E12.6 is enriched in germ line cells, intestine, and I5, DA, SAB, and retrovesicular ganglia.
T05E12.6	T05E12.6 is an ortholog of human EPHX1 (epoxide hydrolase 1); microarray and RNA sequencing studies indicate that T05E12.6 is regulated by heme, diallyl trisulfide, tunicamycin, D-glucopyranose, D-glucose, deoxyglucose, atrazine, fluoranthene, Cry5B, chlorpyrifos, diazinon, quercetin, humic substances, colistin, adsorbable organic bromine compounds, sirolimus, and Ag nanoparticles; RNA sequencing and microarray studies indicate that T05E12.6 is enriched in germ line cells, intestine, and I5, DA, SAB, and retrovesicular ganglia.
acdh-1	acdh-1 encodes a short-chain acyl-CoA dehydrogenase; ACDH-1 is predicted to be a mitochondrial enzyme that catalyzes the first step of fatty acid beta-oxidation and thus plays a key role in energy production; steady-state acdh-1 mRNA levels are controlled by the MDT-15 mediator complex subunit; in addition, gene expression studies indicate that acdh-1 expression is upregulated in daf-2 mutant animals but downregulated in fasted animals; loss of acdh-1 activity via RNAi can shorten the lifespan.

Information taken from Wormbase (Wormbase.org). For additional details and citations, please refer to Wormbase.

**Table 4:** Descriptions of the cellular functions of genes after exposure to MUA/Au NPs = 3.

Gene expression after exposure to MUA/Au NPs = 3	
***Upregulated genes***	
Y65B4BR.1	Y65B4BR.1 is an ortholog of human PLB1 (phospholipase B1); Y65B4BR.1 is involved in cellular defense response; Y65B4BR.1 is predicted to have hydrolase activity, acting on ester bonds, based on protein domain information.
fil-1	fil-1 is involved in lipid catabolic processes and lipid storage; fil-1 exhibits lipase activity; fil-1 is expressed in the intestine.
clec-174	clec-174 is involved in cellular defense response.
F49C12.7	RNA sequencing and microarray studies indicate that F49C12.7 is regulated by ethanol, 1-methylnicotinamide, methylmercuric chloride, D-glucose, chlorpyrifos, diazinon, humic substances, R24, colistin, dafa #1, and sirolimus; proteomic, tiling array, RNA sequencing, and microarray studies indicate that F49C12.7 is enriched in the germ line, coelomocytes, the intestine and neurons.
cysl-2	cysl-2 encodes a homolog of sulfhydrylases/cysteine synthases.
***Downregulated genes***	
mtl-1	mtl-1 encodes one of two *C. elegans* metallothioneins, small, cysteine-rich metal-binding proteins; MTL-1 functions in metal detoxification, homeostasis and stress adaptation; in addition, mtl-1 plays a role in regulating growth and fertility; mtl-1 is constitutively expressed in the terminal bulb of the pharynx and its expression is induced in larval intestinal cells following exposure to cadmium and heat shock and repressed in response to *P. aeruginosa* infection; intestinal expression is dependent on ELT-2, an intestine-specific GATA-type transcription factor; mtl-1 is upregulated by DAF-16 in daf-2 mutants.
ZK593.3	ZK593.3 is involved in lipid storage.
F44A6.5	F44A6.5 encodes a novel protein conserved among nematodes.
T05E12.6	T05E12.6 is an ortholog of human EPHX1 (epoxide hydrolase 1); microarray and RNA sequencing studies indicate that T05E12.6 is regulated by heme, diallyl trisulfide, tunicamycin, D-glucopyranose, D-glucose, deoxyglucose, atrazine, fluoranthene, Cry5B, chlorpyrifos, Diazinon, quercetin, humic substances, colistin, adsorbable organic bromine compounds, sirolimus, and Ag nanoparticles; RNA sequencing and microarray studies indicate that T05E12.6 is enriched in germ line cells, intestine, and I5, DA, SAB, and retrovesicular ganglia.
T05E12.6	T05E12.6 is an ortholog of human EPHX1 (epoxide hydrolase 1); microarray and RNA sequencing studies indicate that T05E12.6 is regulated by heme, diallyl trisulfide, tunicamycin, D-glucopyranose, D-glucose, deoxyglucose, atrazine, fluoranthene, Cry5B, chlorpyrifos, diazinon, quercetin, humic substances, colistin, adsorbable organic bromine compounds, sirolimus, and Ag nanoparticles; RNA sequencing and microarray studies indicate that T05E12.6 is enriched in germ line cells, intestine, and I5, DA, SAB, and retrovesicular ganglia.

Information taken from Wormbase (Wormbase.org). For additional details and citations, please refer to Wormbase.

## Conclusions

In summary, we have demonstrated the adverse effects of bare Au and MUA-Au NPs on the population, body length and locomotion of worms as well as on axonal neuron growth. MUA played a critical role in tuning the size of Au NPs, leading to differentially observed toxic effects. The internalization and absorption of bare Au and MUA-Au NPs into worm’s tissues and body cavities was confirmed by X-ray and confocal microscopy. Cultured neurons exposed to Au NPs grew shorter axons, which likely caused the impeded worm locomotion behavior. Changes in gene expression accurately reflected changes in our observed worm phenotypes after AU NP exposure. Critical examples are clec-174 (involved in cellular defense), cut-3 (involved in body morphogenesis), dpy-14 (expressed in embryonic neurons) and mtl-1 (functions in metal detoxification and homeostasis). Herein, we established a toxicity assessment system for various engineered nanoparticles in *C*. *elegans* and we recommend verifying these Au NP propensities in biomedical applications.

## Materials and Methods

### Preparation of MUA-Au NPs

HAuCl_4_·3H_2_O and MUA were purchased from Sigma-Aldrich Chemical (St. Louis, MO, USA). Briefly, 0.2 mL of MUA at molar concentrations (relative to Au) of 1:2, 1:1, and 3:1 in anhydrous ethanol were mixed with 0.5 mL of 20 mM HAuCl_4_·3H_2_O, and distilled deionized water was then added to a total volume 10 mL. Next, this precursor solution (with different MUA to Au ratios) was illuminated by an intense X-ray beam running at a constant electron current of 300 mA by a top-up injection every minute (BL01A beamline of the National Synchrotron Radiation Research Center, NSRRC, Hsinchu, Taiwan). The X-ray photon energies ranged from 8–15 keV and were centered at ~12 keV; the dose rate was 4.7*10^5^ Gy s^−1^ ^37^. Small-angle X-ray scattering measurements were taken on the BL23A beamline of the NSRRC with 15 keV of photon energy and a 1820.88 mm specimen-to-detector (MarsCCD 1024 × 1024 pixels) distance on MUA-Au NPs. Zeta potential was used to investigate the agglomeration/aggregation characteristics of bare Au NPs and MUA-Au NPs in S medium.

### Nematode Maintenance and Au NP Exposure

*C*. *elegans* (wild-type strain, N2) was cultured on nematode growth medium (NGM) agar plates seeded with (uracil auxotroph) *E*. *coli* OP50 at 20 °C according to standard methods^19^. P0 synchronized worms were exposed to either bare Au NPs or MUA-coated Au NPs, resulting in different particle sizes prepared in S basal liquid medium (worm liquid growth medium)^38^ for 72 h.

### Au NP Uptake and *In Vivo* X-ray Imaging

To determine whether worms can uptake Au NPs, we exposed *C*. *elegans* wild-type (N2) strains to 100 μL of 0.1 mg/mL bare Au or MUA-Au NPs for 12 h in S medium prior to imaging using an X-ray microscope. Microradiology was implemented with unmonochromatized (white) synchrotron X-rays emitted by the 01-A beamline wavelength shifter of the NSRRC. The photon energies ranged from 4–30 keV with a peak intensity at ~12 keV, and the beam current was kept constant at 360 mA under the top-up operation mode.

### Endpoint Toxicity Assays

Changes in worm populations after exposure to bare Au or MUA-Au NPs were evaluated by counting the number of surviving worms under a dissecting microscope. In all experiments, worms were synchronized by the bleaching method, in which adult hermaphrodite worms were exposed to a “bleach solution” (7 mL of ddH_2_O, 1 mL of 5 M NaOH and 2 mL of 6–12% NaOCl) that disintegrated the cuticle and allowed for egg collection. These eggs were incubated with either bare Au NPs or various MUA-Au NP ratios (resulting in different particle sizes) for 72 h in S medium before calculating the number of offspring worms. The same worms were also employed to measure body length, locomotion and brood size. The worm body length was measured using the open-source NIH imaging software ImageJ (http://rsb.info.nih.gov/ij/). Here, we straightened digital (brightfield) images of (otherwise) bended worms using the ImageJ plugin ‘Straighten Tool’ and then used the ImageJ ‘Line Tool’ to measure the worm’s body length. Changes in locomotion behavior were assessed by counting the worms’ body bends per minute in liquid S medium (worm thrashing assay). To evaluate the worm brood size, individual untreated or treated young adult worms were transferred to an NGM plate seeded with *E*. *coli* OP50 at 20 °C, and the number of progeny was counted after 72 h. Each experiment noted above was independently repeated 5 times for each MUA to Au ratio used.

### Isolation and Culturing of Primary *C*. *elegans* Neurons

Based on protocols reported by Christensen *et al*.^39^ and Strange *et al*.^40^, primary *C*. *elegans* neurons were isolated and cultured. All worms express the pan-neuronal marker pUNC-104::UNC-104::mRFP^41^ to improve the visualization of neuronal structures (soma, axons and dendrites) under a confocal microscope. In brief, eggs isolated from these worms were collected from gravid hermaphrodites using the abovementioned bleaching method. The enzyme chitinase (Sigma, 1 U/mL egg solution) was then added to the egg suspensions to dissolve the compact egg shell. Exposed embryonic cells were subsequently plated on lectin-coated glass-bottom Petri dishes using 2 mL of Leibovitz’s L-15 medium (containing 10% FBS and antibiotics) mixed with 100 μL of 0.1 mg/mL bare Au NPs or MUA-Au NPs. After 3 days of incubation, the neurons were fully developed and could be imaged and analyzed. To measure axonal length, we first straightened the digital images of axons using the ImageJ plugin ‘Straighten Tools’ and then used the ‘Line Tool’ to measure their lengths. Each experiment was independently repeated 3–5 times for each MUA to Au ratio used.

### MTT Assay

The viability (survival) of cultured primary *C*. *elegans* neurons was determined using the indicator 3-(4,5-dimethyl-thiazol-2-yl)-2,5-diphenyltetrazolium bromide (MTT assay). Here, neurons were cultured in 24-well culture dishes coated with lectin at a density of 10^5^ cells/mL in 1000 μL of L-15 medium. Next, 10 μL of MTT was added to each well, and neurons were exposed to various ratios of MUA-Au NPs (bare, 0.5, 1, or 3) for 3 days. After 4 h of incubation, 10 μL of HCl and isopropanol were added to each well and shaken for 5 minutes. The optical density (OD) of each well was then measured at 570 nm with an ELISA microplate reader (VICTOR X3, Perkin Elmer). The OD value was calculated by the following equation: S = (OD_treated sample_ − OD_blank_)/(OD_control sample_ − OD_blank_) * 100%.

### Microarray analysis

We employed DNA microarray analysis using the ‘Affymetrix *C*. *elegans* gene chip’ (Thermo Fisher Scientific Inc., Santa Clara, CA, USA) covering 22,548 transcripts. In short, total RNA was isolated from synchronized worms grown in liquid culture (exposed to either 0.1 mg/mL bare Au NPs or to different ratios of MUA-Au NPs) using TRIZOL-based standard protocols^42^. The quality of RNA was monitored using OD measurements, and an OD 260/280 ratio >1.9 was deemed adequate. Subsequent fluorescence labeling was performed using the Genispehere 3DNA Array350 kit (Genisphere Inc., Hatfield, PA, USA). The gene chip was hybridized to cDNA overnight, washed stringently to remove nonspecifically bound probe, and then poststained with fluorescent dendrimers. After posthybridization, a GenePix scanner and GenePix Pro 4.0 image analysis software (Molecular Devices, Sunnyvale CA, USA) were used to determine the fluorescence intensities. We considered hybridization spots positive if the signal intensity was at least two times that of the background in at least one channel in half of the replicates. Per-chip normalization was performed by dividing the expressed genes by the median of two housekeeping control genes, β-tubulin and cyclophilin. For data mining and statistical analysis, we used the Rosetta Resolver System (Rosetta Biosoftware, Seattle, WA, USA)^43^ as well as Transcriptome Analysis Console (TAC) software^44^ (Affymetrix, Waltham, MA, USA). To understand the cellular functions of the genes, we employed Wormbase (wormbase.org)^45^, which annotates and describes all known *C*. *elegans* genes, i.e., gene expression patterns in various worm tissues, the relationships between different genes (interactome), the functions of cellular pathway genes and homologies to other organisms. All microarray experiments were carried out at the NHRI Microarray Core Laboratory (Zhunan, Taiwan), which houses an Affymetrix GeneChip system consisting of the GeneChip Hybridization Oven 640, GeneChipFluidics Station 450, and GeneChipScanner 3000 (Affymetrix).

### Statistical Analysis

Significant differences between experimental groups were determined using Student’s t-tests (employing Microsoft Office Excel’s ‘Data Analysis’ tool). Three to five independent experiments were performed for each experimental condition, and all data are expressed as the mean ± STD (obtained from at least three independent experiments). P values are represented as *p < 0.05 and **p < 0.01.

## Electronic supplementary material

Supplementary Information

3D confocal images of young adult worm's intestine.
