# Supplementary material for: Toxic Effects of Size-tunable Gold Nanoparticles on Caenorhabditis elegans Development and Gene Regulation
Source: Sci Rep. 2018 Oct 15;8:15245. doi: 10.1038/s41598-018-33585-7 (PMC6189128; doi:10.1038/s41598-018-33585-7)
Supplement: Supplementary file 1 — Supplementary Information [file 41598_2018_33585_MOESM1_ESM.docx]

Supplementary Information

**Toxic Effects of Size-tunable Gold Nanoparticles on *Caenorhabditis elegans* Development and Gene Regulation**

**Chun-Chih Hu^1^, Gong-Her Wu^2^, Sheng-Feng Lai^3^, Muniesh Muthaiyan Shanmugam^2^, Y. Hwu^3^, Oliver I. Wagner^2,*^, and Ta-Jen Yen^1,*^**

^1^Department of Materials Science and Engineering, National Tsing Hua University, Hsinchu 30013, Taiwan

^2^Department of Life Science and Institute of Molecular & Cellular Biology, National Tsing Hua University, Hsinchu 30013, Taiwan

^3^Institute of Physics, Academia Sinica, Taipei 115, Taiwan

*Corresponding authors:Ta-Jen Yen, PhD, Professor, Department of Materials Science and Engineering

Oliver I. Wagner, PhD, Professor, Department of Life Science

National Tsing Hua University

101, Sec. 2, Kuang-Fu Road

Hsinchu 30013

Taiwan R. O. C.

Emails: tjyen@mx.nthu.edu.tw, owagner@life.nthu.edu.tw (to whom correspondence should be addressed)

Supplementary Information

**Au NP Labeling with FITC−APTMS.** Fluorescein isothiocyanate modified with 3-aminopropyltrimethoxysilane (FITC−APTMS) was utilized to make MUA-Au or bare Au NPs visible in worm bodies. For fluorescence imaging of FITC coated Au NPs and bare Au NPs, Carl Zeiss LSM 780 microscope accompanied by ZEN software was used. Low magnification images showing full worm were obtained with 10X objection lens using a gain value of 500 for 488 nm wavelength laser, however high magnification worm images were obtained with 63X objective lens using a gain value of 700 for 488 nm wavelength laser and 200 gain value for bright-field. Brightness and contrast were adjusted in a similar manner for all low magnification images, FITC high magnification images and Bright-Field/merged images. Fluorescent intensity in the worm tissue was measured using ImageJ after background subtraction. The integrated density measured from ImageJ was normalized for the area measured and represented as fluorescent intensity per 100 µm^2^ area of worm tissue. One way ANOVA with Fisher’s LSD test was used to compare the study groups.

***In vivo* imaging of FITC-labeled Au NPs.** We utilized confocal microscopy to image the absorption of FITC-labeled Au NPs from the intestines into body tissues and subsequently into body cavities of *C. elegans*. Figure S1a shows that MUA-Au and bare Au NPs can cross the intestine and reach several of the tissues and body cavities. Further, uptake of MUA-Au NPs into the worm’s tissues is more obvious when compared to that of bare Au NPs (Figure S1b). Note that in control worms (Figure S1a, no Au NPs treatment) autofluorescence can be seen derived from lipofuscin granules.^1-3^ Because autofluorescence is a mixture of various colors (green, red, yellow etc.), we confirmed that in treated animals both can be seen: autofluorescence as well as sharp, solitary green FITC signals. From these results we conclude that uptake of Au NPs into tissues may alter gene expression (Table 2-4) and concomitantly worm behavior.
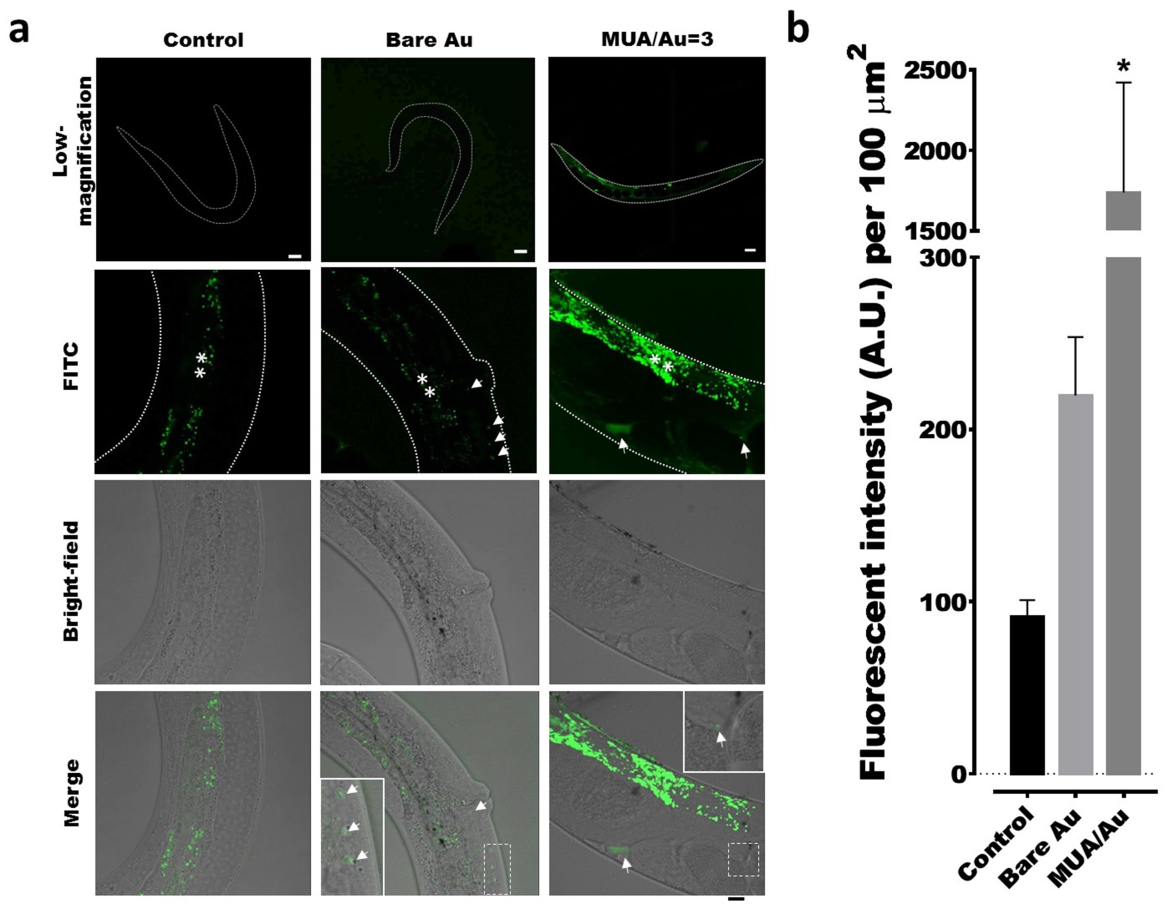


Figure S1. (a) Confocal images of young adult worms treated with or without FITC-Au NPs revealing FITC signal emanating from the worm’s body tissues. (b) Fluorescent intensity quantification in body tissues (excluding the intestine) of *C. elegans* in different study groups. ** Refers to the location of the worm’s intestine (please note the auto-fluorescence derived from ingested OP50 bacteria in control group). White arrows indicate FITC-Au NPs in the body tissues and body cavities. White dashed lines indicate the boundary of the worm. Dashed box represents the digitally magnified inlet. Scale bar is 50 µm (inset scale bar is 10 µm). One-way ANOVA with Fisher’s LSD test, *p < 0.05. Error bar ± SEM. We confirmed that in the treatment group fluorescence derived from both auto-fluorescence (which is a mixture of various colors such as red, green and yellow) as well as FITC (which derived form a sharp, solitary green fluorescence signal). A.U. – arbitrary unit.

**Endocytosis of MUA-Au and bare Au NPs in neurons.** To verify whether Au NPs can be directly uptaken into neurons, primary neuronal cells (isolated form the worm’s embryos) were exposed to either MUA-Au or bare Au NPs. It is evident from Figure S2 that cultured neurons can also uptake FITC-labeled Au NPs (arrows in Figure S2d), which is likely the cause of changes in worm’s locomotion (Figure 4c) based on axonal development defects (Figure 5b).


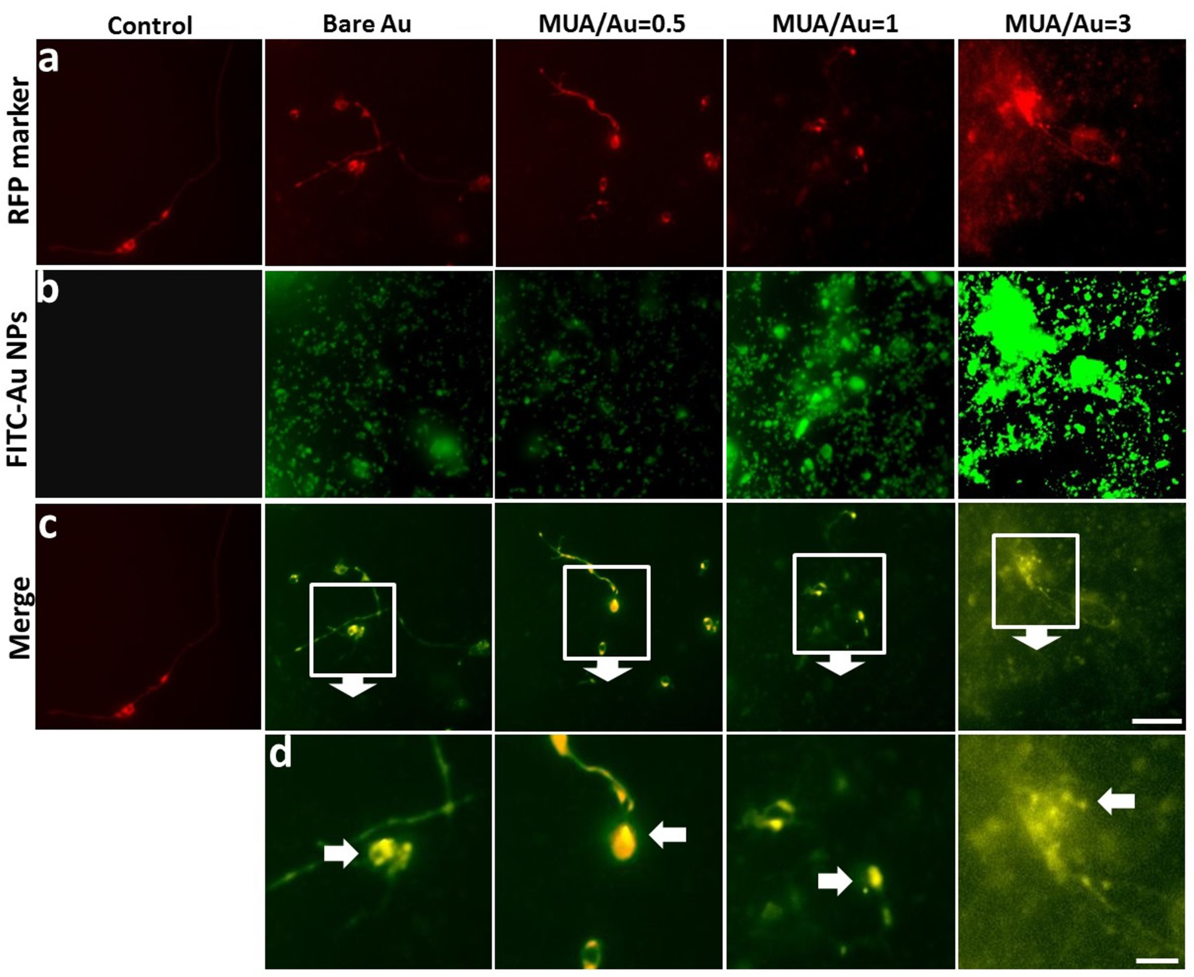


Figure S2. Confocal images of neuronal cells (expressing red fluorescent neuronal marker) treated with FITC-labeled Au NPs with different MUA/Au ratios. (a) Red channel reveals neuronal morphology. (b) Green channel reveals FITC−Au NPs. (c) Merged images allow for the detection of colocalization events. (d) Magnifications of insets as shown in (c). The arrows point to incorporated Au NPs. Scale bars: (a−c) 20 μm and (d) 10 μm.

MOVIE 1. 3D confocal images of young adult worm’s intestine.

References

1. Clokey, G.V. & Jacobson, L.A. The autofluorescent "lipofuscin granules" in the intestinal cells of Caenorhabditis elegans are secondary lysosomes. *Mech Ageing Dev* **35**, 79-94 (1986).

2. Forge, T.A. & Macguidwin, A.E. Nematode autofluorescence and its use as an indicator of viability. *J Nematol* **21**, 399-403 (1989).

3. Pincus, Z., Mazer, T.C. & Slack, F.J. Autofluorescence as a measure of senescence in C. elegans: look to red, not blue or green. *Aging (Albany NY)* **8**, 889-898 (2016).
